# Multiple Myeloma Cells Shift the Fate of Cytolytic ILC2s Towards TIGIT-Mediated Cell Death

**DOI:** 10.3390/cancers17020263

**Published:** 2025-01-15

**Authors:** Fabiana Drommi, Alessia Calabrò, Gaetana Pezzino, Grazia Vento, Josè Freni, Gregorio Costa, Riccardo Cavaliere, Irene Bonaccorsi, Alessandro Allegra, Guido Ferlazzo, Claudia De Pasquale, Stefania Campana

**Affiliations:** 1 Laboratory of Immunology and Biotherapy, Department Human Pathology in Adulthood and Childhood “Gaetano Barresi”, University of Messina, 98125 Messina, Italy; drommifabiana@gmail.com (F.D.); calabroalessia@hotmail.it (A.C.); tpezzino@unime.it (G.P.); gregorio.costa@unime.it (G.C.); rcavaliere@unime.it (R.C.); ibonaccorsi@unime.it (I.B.); cdepasquale@unime.it (C.D.P.); scampana@unime.it (S.C.); 2 Department of Experimental Medicine (DIMES), University of Genoa, 16132 Genova, Italy; grazia.vento@edu.unige.it; 3 Laboratory of Histology, Department of Biomedical, Dental, Morphological and Functional Imaging Sciences, University of Messina, 98125 Messina, Italy; jofreni@unime.it; 4 Clinical Pathology Unit, University Hospital Policlinico “G. Martino”, 98125 Messina, Italy; 5 Division of Hematology, Department of Human Pathology in Adulthood and Childhood “Gaetano Barresi”, University of Messina, 98125 Messina, Italy; alessandro.allegra@unime.it; 6 Unit of Experimental Pathology and Immunology, IRCCS Ospedale Policlinico San Martino, 16132 Genova, Italy

**Keywords:** group 2 innate lymphoid cells (ILC2s), multiple myeloma (MM), granzyme B (GZMB), DNAM-1, TIGIT, human

## Abstract

Background: Growing evidence attests to the multifaceted roles of group 2 innate lymphoid cells (ILC2s) in cancer immunity. They exhibit either pro- or anticancer activity depending on tumor type but their function in Multiple Myeloma (MM) is still not elucidated. Methods: The bone marrow (BM) and peripheral blood (PB) of patients (pts) with MM or precancerous conditions were collected, and specific properties of ILC2 subsets were assessed by flow cytometry. Results: By dissecting ILC2s according to c-Kit marker, we observed that NKp30 and NKG2D were mainly confined to c-Kit^hi^ ILC2s, while levels of DNAM-1 was significantly higher in fully mature c-Kit^lo^ cells. Among the total MM-associated ILC2s (MM-ILC2s), we observed a significant increase in c-the Kit^lo^ subset, but the expression of DNAM-1 in these cells was significantly reduced, especially in BM. Interestingly, MM-ILC2s from PB expressed granzyme B (GZMB), but its expression was impaired in BM-ILC2s. Accordingly, MM cells were susceptible to killing by MM-ILC2s derived from PB while eluding ILC2 surveillance in BM. Indeed, in MM-ILC2s derived from BM, the downregulation of DNAM-1 is accompanied by the upregulation of TIGIT, which mediate cell death in ILC2s upon recognition of the cognate ligands expressed by MM cells. These ILC2 changes appeared in clinical precursor conditions and eventually accumulated with disease progression. Conclusions: MM-ILC2s can act as cytolytic immune effectors that are fully competent in PB. However, MM cells shift ILC2 fate towards cell death in BM via the upregulation of TIGIT, thereby representing a potential therapeutic target to restore ILC2 antitumor activity.

## Simple Summary

Multiple myeloma (MM) is a plasma cell malignancy that develops in the bone marrow (BM) and the second-most-common blood cancer. Group 2 innate lymphoid cells (ILC2s) are emerging cytolytic immune effectors showing potent antitumor activity. In MM patients, ILC2s from peripheral blood possessed a cytotoxic arsenal, enabling them to kill MM cells via DNAM-1. In contrast, ILC2 resident in BM did not exert anti-myeloma activity, and instead became more susceptible to apoptosis. Specifically, MM cells led to ILC2 cell death by inducing and engaging the inhibitory receptor TIGIT. Collectively, ILC2s from MM patients displayed an imbalance in the DNAM-1/TIGIT axis, resulting in the conversion from a cytolytic to an exhausted profile. These findings support the employment of anti-TIGIT mAb to restore the survival and antitumor effects of ILC2s in MM.

## 1. Introduction

Group 2 innate lymphoid cells (ILC2s) are innate immune sentinels activated by alarmin cytokine cues such as IL-25 and IL-33 and play critical roles in immunity, particularly early on after infection with parasites [1,2]. At mucosal barriers, they are important regulators of tissue homeostasis and repair by fine-tuning innate–adaptive immune cell responses [3]. ILC2s are also involved in the regulation of cancer growth either by directly interacting with cancer cells or by interacting with other immune or non-immune cells [4]. By abundantly secreting type 2 cytokines, ILC2s are strongly associated with a microenvironment promoting tumor growth and blocking antitumor immunity [5].

In acute promyelocytic leukemia (APL), IL-13-producing ILC2s participate in the establishment of a pro-tumor microenvironment by activating monocytic myeloid-derived suppressor cells (M-MDSCs) [5]. This tolerogenic pathway is promoted by the interaction of CRTH2 and NKp30 with high levels of tumor-derived PGD2 and B7-H6, respectively [6]. In line with these findings, in patients (pts) bearing prostate and bladder cancer, a positive correlation between ILC2s and M-MDSCs has been reported [6,7]. In a mammary cancer model, tumor growth was sustained by ILC2/M-MDSC/Tregs immunosuppressive axis [8]. On the other hand, ILC2s can contribute to the rejection of tumors in mouse models of melanoma, pancreatic cancer and CRC [9,10,11]. In particular, their antitumor activity relies on the capability to enhance the responses of cytotoxic T cells and eosinophils [9,10]. In a murine lymphoma model, ILC2s were able to produce a large amount of the CXCR2 ligands CXCL1 and CXCL2, and interaction with a CXCR2-expressing tumor was shown to induce massive tumor-specific apoptosis in a CXCR2-specific way [12]. In this regard, it has been recently described that, similar to NK cells, human ILC2s can acquire cytolytic abilities specifically against tumor cells [13]. Mechanistically, human ILC2s can produce the cytotoxic molecule Granzyme B (GZMB) and directly lyse tumor cells by inducing pyroptosis and/or apoptosis, which is governed by DNAM-CD112/CD155 interaction [13]. The recent finding that ILC2s represent a member within the family of cytolytic immune effector cells prompted us to investigate their cytolytic potential in Multiple Myeloma (MM), a plasma cell malignancy that develops in the bone marrow (BM) and is often preceded by asymptomatic precursor conditions such as monoclonal gammopathy of undetermined significance (MGUS) and smoldering MM (sMM) [14]. By analyzing both peripheral blood (PB) and BM-resident ILC2s in newly diagnosed MM pts, we observed a significant downregulation of DNAM-1 expression that reached the lowest level in BM. Moreover, in MM-ILC2s derived from BM, the downregulation of DNAM-1 was accompanied by the upregulation of TIGIT, which had already appeared in clinical precursor conditions. Functionally, ILC2s from PB-MM largely expressed GZMB and exerted cytolytic activity toward MM cells via DNAM-1, while in BM, engagement of TIGIT resulted in ILC2 cell death, thereby suggesting TIGIT as a therapeutic target to restore ILC2 antitumor activity.

## 2. Material and Methods

### 2.1. Sample Collection

Peripheral blood (PB) and bone marrow (BM) samples were collected from patients newly diagnosed with monoclonal gammopathy of undetermined significance (MGUS) (*n* = 4), smoldering Multiple Myeloma (sMM) (*n* = 4) and untreated MM (pts) (*n* = 20). The PB of healthy donors (HD) (*n* = 20) and BM (*n* = 4) was used as a control. The patients’ clinical parameters are illustrated in Table S1. All the pts were admitted to the Hematology Unit of University Hospital Policlinico G. Martino, Messina. The study was approved by the institutional ethics committees and all participants gave written informed consent according to the Declaration of Helsinki (ID number 04/19 in 11 January 2019).

### 2.2. Cell Isolation and Phenotypical Analysis

PB and BM were processed as previously described [15]. Briefly, mononuclear cells from PB and BM were isolated by Ficoll Hypaque density gradient centrifugation (30 min, 25 °C, 400× *g*) (VWR International, Radnor, PA, USA) and then immediately analyzed or stored at −80 °C for later analysis. Cell populations of interest from HDs or MM pts were isolated by using the following gating strategies: ILC2s as 7-AADneg CD45+ LINneg (CD3, CD19, CD14, NKp80) CD127+ c-Kit^hi/lo^ CRTH2+ [16]; freshly primary MM cells from BM as 7-AADneg CD45neg CD138+CD38+. For surface staining, cells were incubated with specific mAbs for 15 min at room temperature and then PBS-washed. For the intracellular staining of IL-10 and IL-13, following stimulation, and granzyme B (GZMB) production, cells were surface-stained as above described and then fixed in 1% paraformaldehyde for 20 min on ice in the dark, permeabilized with saponin 0.1% in PBS and stained with anti-IL10, anti-IL13 and anti-GZMB mAbs for 45 min at room temperature [17]. Cells were washed twice with PBS and then analyzed. Intranuclear staining was performed for the detection of GATA-3 transcription factor on ILC2s isolated from PB and BM of HDs and MM pts according to the manufacturer’s protocol (Transcription Factor Staining Buffer Set, Miltenyi, Bergisch Gladbach, Cologne, Germany). ILC2s from the PB and BM of MM pts were stained with Annexin V-FITC for 30 min at 4 °C in the dark and then analyzed by flow cytometry.

### 2.3. Cell Culture Assay

To assess GZMB expression, ILC2s isolated from HDs were cultured, with P815 cells coated with specific antibodies. Briefly, for Ab coating, P815 cells (ATCC TIB-64™) were propagated in a RPMI complete medium and incubated at 1 × 10^6^ cells/mL for 30 min at room temperature with anti-DNAM-1 (clone F22). Following PBS washing, ILC2s were cultured with coated P815 at a ratio of 1:1 in round-bottom 96-well plates for 48 h. Alternatively, HD-ILC2s were stimulated with PMA (10 ng/mL) plus Ionomycin (500 ng/mL) for 3 h and then assessed for GZMB expression. In addition, GZMB was evaluated on HD and PB-MM ILC2s following co-culture with primary MM cells at a ratio of 1:1 for 3 days. For blocking experiments, PB-ILC2s from HDs and MM pts were treated for 30 min with anti-DNAM-1-blocking antibody (clone F5) or corresponding human IgM as an isotype control before culture with MM cells. PB-ILC2s from HDs and MM pts were co-cultured with primary MM cells at a ratio of 1:1, and expression of DNAM-1 and TIGIT was evaluated daily for 5 days. To assess IL-10 production by ILC2s from BM-MM pts or IL-13 by ILC2s from PB-HD, cells were stimulated in the presence of PMA and Ionomycin for 4 h; monensin and brefeldin (2 μmol/L and 10 μg/mL, respectively; Sigma-Aldrich, St. Lous, MO, USA) were added in the last 3 h. Following incubation, cells were intracellularly stained for IL-10 or IL-13 production as described above.

### 2.4. Cell Killing Assay

To assess ILC2-mediated cytotoxicity against primary MM cells, we performed a flow cytometric and non-radioactive target-based assay. Briefly, ILC2s isolated from the PB-HD, PB and BM of MM pts were co-cultured with primary MM cells at an effector/target ratio of 1:1. DNAM-1-blocking mAb (clone F5) was also added to the PB-MM ILC2 and MM cell culture. The target cells were pre-labeled with a red fluorescent dye, PKH-26 (Sigma-Aldrich, St. Lous, MO, USA), according to the manufacturer’s instructions, to allow for their discrimination from the effector cells. After 48 h, killed target cells were identified through 7-AAD staining, which specifically permeated dead cells. Data analysis was performed by gating on the PKH-26+ target cells, followed by the analysis of the 7-AAD+ subpopulation. Target cell death was calculated based on the following ratio: [(% sample cytotoxicity − % spontaneous cytotoxicity)/(% total cytotoxicity − % spontaneous cytotoxicity)]. To evaluate ILC2 apoptosis, purified BM-ILC2s were co-cultured with autologous purified MM cells at an E:T ratio of 1:1 in the presence or absence of 5 μg/mL of anti-TIGIT neutralizing antibody (Tiragolumab Cat. No.: HY-P9986, MedChem Express, Monmouth Junction, NJ, USA) or corresponding human IgG1 kappa as an isotype control. Following 48 h of incubation, apoptotic ILC2 cells were evaluated using the Annexin V-FITC Apoptosis detection kit according to the manufacturer’s instructions (Beckman Coulter, Brea, CA, USA) within 7-AAD+/neg cells. To examine MM cell death, tumor cells were co-cultured with or without ILC2s at an E:T ratio of 1:1 in the presence or absence of 5 μg/mL of anti-TIGIT neutralizing antibody (Tiragolumab Cat. No.: HY-P9986, MedChem Express, Monmouth Junction, NJ, USA) or corresponding human IgG1 kappa as an isotype control. After 48 h, MM cell viability was evaluated as a percentage of 7-AAD+ cells within CD45negCD38+CD138+ cells.

### 2.5. Flow Cytometry

The mAbs used in this study are summarized in Table S2. All supernatant mAbs were produced at UO Patologia e Immunologia Sperimentale at IRCCS Ospedale Policlinico San Martino of Genoa. Sample acquisition was performed on FACSCantoII or FACSymphony (BD Biosciences, Franklin Lakes, NJ, USA) flow cytometers, and cell sorting was performed on the FACSAria II cell sorter (BD Biosciences). Data were acquired by FACS Diva (BD Biosciences) and analyzed by FlowJo version VX (Tree Star Inc., Oakland, CA, USA) software.

### 2.6. Statistical Analysis

Depending on the data, either a paired Student’s *t*-test or an ANOVA test was applied to evaluate statistical significance. *p*-Values lower than 0.05 were considered statistically significant (* *p* < 0.05; ** *p* < 0.01; *** *p* < 0.001). Statistics were calculated using GraphPad Prism 4 software.

## 3. Results

### 3.1. ILC2s Are Reduced in MM pts and Enriched in c-Kit^lo^ Subset

**Figure 1:**
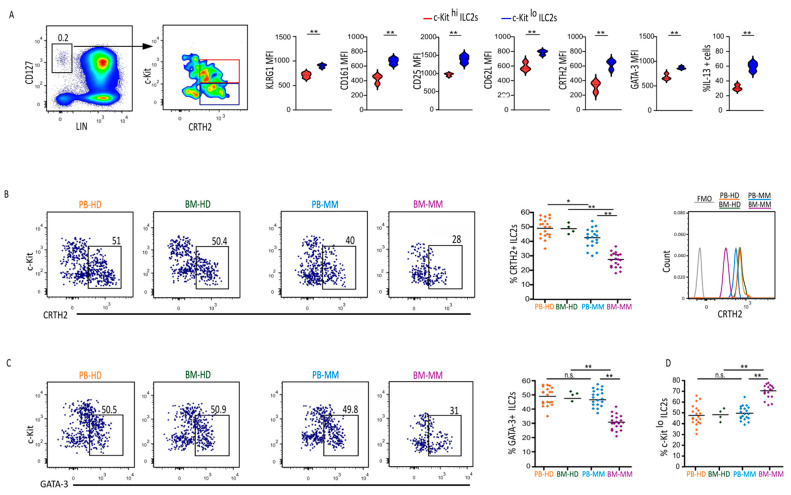
Frequency and subset distribution of ILC2s in MM pts: (**A**) Gating strategy to identify c−Kit^hi^ and c−Kit^lo^ ILC2 subsets (7−AADneg, CD45+ LIN neg CD127+ c−Kit ^hi/lo^ CRTH2+ GATA-3+) from PB−HDs. Violin plots represent the expression of the KLRG1, CD161, CD25, CD62L, CRTH2 and GATA−3 and IL−13 produced in c−Kit^hi/lo^ ILC2s from PB−HDs, ** *p* < 0.01 (*n* = 20). (**B**) Representative dot plots and statistical analysis showing the frequency of CRTH2+ILC2 in the PB (*n* = 20) and BM (*n* = 4) of HDs and MM pts (*n* = 20). Scatter plots showing the percentage ±SEM of CRTH2+ ILC2s. * *p* < 0.05, ** *p* < 0.01. Histograms showing the MFI of CRTH2 in the PB (*n* = 20) and BM (*n* = 4) of HDs and MM pts (*n* = 20). Fluorescent minus one (FMO) staining was used as a negative control. (**C**) Representative dot plots and statistical analysis showing the frequency of GATA−3+ ILC2s from the PB (*n* = 20) and BM (*n* = 4) of HDs and MM pts (*n* = 20). ** *p* < 0.01; n.s., not significant. (*n* = 20). (**D**) Scatter plots showing the percentage of c−Kit^lo^ ILC2s from the PB (*n* = 20) and BM (*n* = 4) of HDs and MM pts (*n* = 20). ** *p* < 0.01; n.s., not significant.

ILC2s have been classically identified as LIN neg CD127+ CRTH2+ cells showing heterogeneous expression of the surface marker c-Kit [18,19]. In humans, c-Kit is a useful marker, able to distinguish two functionally distinct populations of ILC2s [20]: c-Kit^lo^ ILC2s are fully mature ILC2s as they are the most potent type 2 cytokine producers, while c-Kit^hi^ ILC2s show more plastic behavior, converting to an ILC3 and ILC1-like phenotype depending on the cytokine milieu of the microenvironment [19]. Supporting this evidence, the expression level of ILC2 common markers including KLRG1, CD161, CD25, CD62L, CRTH2, and the transcription factor GATA-3 was higher in the c-Kit^lo^ subpopulation that responded with a higher production of IL-13 upon PMA/Ionomycin stimulation, as compared with that of c-Kit^hi^ ILC2s (Figure 1A). Although changes in ILC2s in human leukemia have been described [6], data related to modifications of ILC2s in MM pts are still lacking. To better define the distribution and the specific properties of ILC2 subsets during MM disease, we analyzed their features in the BM and PB of newly diagnosed, not treated, MM pts. We first determined the frequency of ILC2s in both PB and BM compartments and compared it with those in healthy donors (HD). We found a lower amount of ILC2s in PB, and this decrease was more pronounced in the BM of MM patients (Figure 1B). However, we observed a downregulation of the canonical marker CRTH2 in MM patients (Figure 1B), which commonly occurred upon activation and might have potential implications for detecting ILC2s. To overcome this issue, we instead used the GATA-3 marker to assess ILC2 quantification, confirming the reduction in these cells in the BM but not in the PB of MM patients (Figure 1C) and highlighting that CRTH2 fails in identifying ILC2s in this context. Concerning subset distribution, while, on average, c-Kit^hi^ ILC2 dominated (50% to 60%) when compared to c-Kit^lo^ cells (40% to 50%) in the blood of healthy donors (HDs) [20], this ratio changed in MM patients, where the percentage of c-Kit^lo^ increased significantly in the BM (65% to 75%) (Figure 1D and Figure S1B).

### 3.2. ILC2s from PB but Not BM of MM pts Produce Granzyme B and Kill MM Cells via DNAM-1

**Figure 2:**
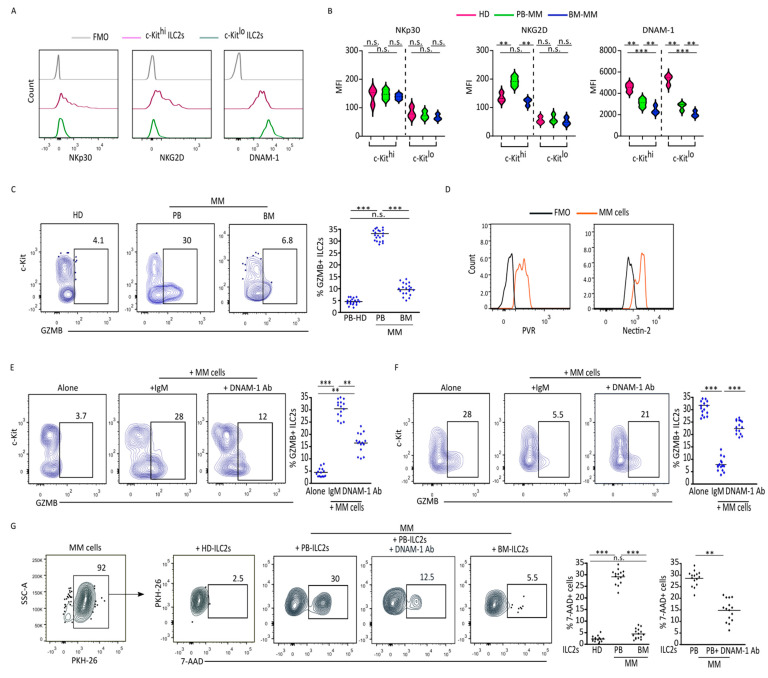
PB−ILC2s from MM pts express GZMB and lyse MM cells upon DNAM−1 engagement: (**A**) Representative histograms representing expression of NKp30, NKG2D and DNAM−1 on c−Kit^hi^ and c−Kit^lo^ ILC2s from PB−HDs. FMO staining was used as a negative control. (**B**) Violin plots represent the expression of NKp30, NKG2D and DNAM−1 on c−Kit^hi^ and c−Kit^lo^ ILC2s from HDs and MM pts (PB and BM). ** *p* < 0.01; *** *p* < 0.001; n.s., not significant. (*n* = 20). (**C**) Intracellular expression of GZMB was assessed on ex vivo ILC2s from HDs and MM pts (PB and BM). Scatter plots represent frequency ±SEM of GZMB+ ILC2s. *** *p* < 0.001. n.s., not significant. (*n* = 20). (**D**) PVR and Nectin−2 expression assessed on MM cells from BM−MM. FMO staining was used as negative control. (**E**) Representative dot plots and relative statistical analysis of GZMB expression in ILC2s from PB−HDs following 3 days of culture with MM cells in presence of DNAM−1-blocking antibody or IgM isotype as control. Scatter plots represent frequency ± SEM of GZMB+ ILC2s. ** *p* < 0.01; *** *p* < 0.001. (*n* = 15). (**F**) Representative dot plots and relative statistical analysis of GZMB expression in ILC2s from PB−MM following 3 days of culture with autologous MM cells in presence or absence of DNAM−blocking mAb. Scatter plots represent frequency ± SEM of GZMB+ ILC2s. *** *p* < 0.001. (*n* = 15). (**G**) Percentage of MM dead cells identified by 7−AAD, following 48 h of co−culture with ILC2s isolated from HDs or MM pts (PB and BM). DNAM−1−blocking mAb was added into co-culture where indicated. Scatter plots represent frequency ±SEM of 7−AAD+ MM cells. ** *p* < 0.01; *** *p* < 0.001. n.s., not significant; (*n* = 15).

The recent findings that ILC2s can express activating receptors involved in the recognition of tumor cells, such as NKp30, NKG2D, and DNAM-1 [6,13], prompted us to investigate their expression in ILC2s from MM patients. According to the expression of c-Kit, we observed that, in ILC2s from HDs, NKG2D and NKp30 were considerably expressed in c-Kit^hi^ ILC2s while they were largely absent in c-Kit^lo^ (Figure 2A). On the contrary, DNAM-1 was present on all ILC2s, being observed at significantly higher levels on c-Kit^lo^ ILC2s (Figure 2A), suggesting that this receptor could be preferentially involved in the recognition of tumor cells by mature ILC2s. In MM patients, ILC2s from PB but not from BM exhibited a higher expression of NKG2D mainly confined to c-Kit^hi^ cells, whose frequency decreased in MM patients, and no difference in NKp30 expression was found independently of the MM pts compartmentally analyzed (Figure 2B). On the other hand, the expression level of DNAM-1 was lower in ILC2s from MM, reaching the lowest level in the c-Kit^lo^ subset present in the BM (Figure 2B). It has been recently reported that the engagement of DNAM-1 on ILC2s resulted in its downregulation and the induction of cytotoxic effector molecule granzyme B (GZMB) [13]. Following DNAM-1-specific triggering on HD-ILC2s, we observed that GZMB was predominantly expressed by c-Kit^lo^ rather than by c-Kit^hi^ (Figure S2A). Similarly, after PMA/Ionomycin stimulation, HD-ILC2s were able to acquire GZMB mainly in the c-Kit^lo^ subset (Figure S2B). Given the reduction in DNAM-1 in the ILC2s of MM patients, we investigated their expression of GZMB and, interestingly, we found that the ILC2s of the PB but not BM expressed a considerable level of this cytotoxic molecule (Figure 2C). The ligands for DNAM-1 (CD155, CD112) are highly expressed in primary MM cells (Figure 2D), and co-culture with HD-ILC2s resulted in the production of GZMB by ILC2s, which was abrogated in the presence of the DNAM-1-blocking antibody (Figure 2E). On the other hand, co-culture of MM cells with ILC2s from the PB of MM pts resulted in a significant DNAM-1-dependent downregulation of GZMB, reminiscent of what was observed in the BM (Figure 2F). This pattern suggests that a strong and prolonged interaction with MM cells, mainly occurring in the BM, could impair the cytolytic activity of ILC2s. Accordingly, ILC2s from PB-MM but not BM-MM were able to kill autologous plasmablasts, and their cytolytic ability depended, as expected, on DNAM-1 engagement (Figure 2G). Overall, these data indicate that the ILC2s from the PB, but not from the BM, of MM pts retained cytolytic ability against MM cells, which is mediated by DNAM-1-engagement and subsequent GZMB release.

### 3.3. MM Cells Induce TIGIT-Mediated Cell Death of BM ILC2s

**Figure 3:**
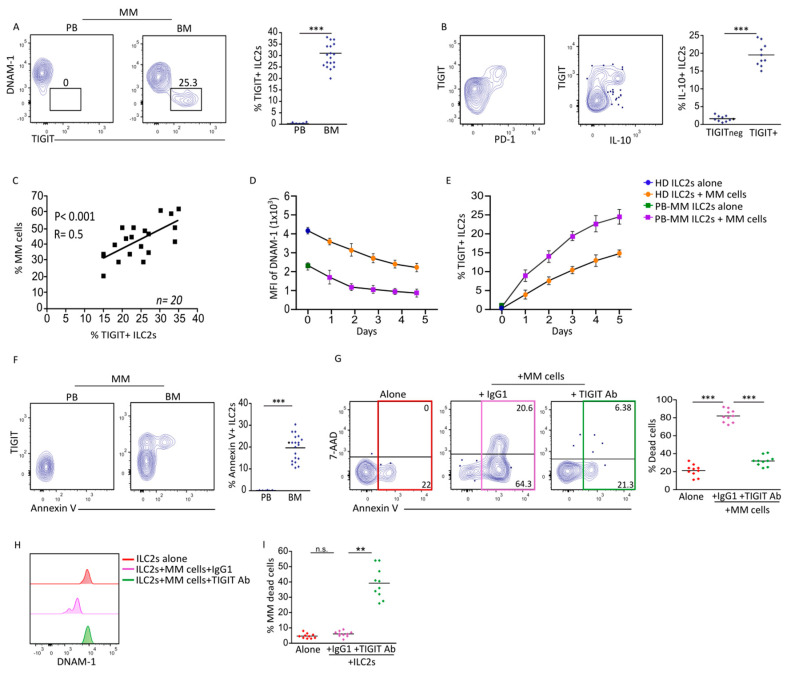
MM cells induce TIGIT−mediated ILC2 cell death: (**A**) Representative dot plots and relative statistical analysis showing expression of DNAM−1 and TIGIT on ILC2s from PB and BM of MM pts. Scatter plots indicate percentage ± SEM of TIGIT+ ILC2s. *** *p* < 0.001. (*n* = 20). (**B**) Flow cytometry analysis showing steady-state expression of PD−1 and IL−10 production following PMA/Ionomycin stimulation on BM−MM ILC2s. Scatter plots indicate percentage ± SEM of IL−10-producing ILC2s. *** *p* < 0.001. (*n* = 10). (**C**) Correlation between percentage of MM cells and TIGIT+ ILC2s in BM−MM (*n* = 20). *** *p* < 0.001; R = 0.5. (*n* = 20). (**D**) Graph represents daily expression of DNAM−1 on ILC2s from HDs and PB−MM pts following 5 days of culture with autologous MM cells. (*n* = 10). (**E**) Graph represents daily expression of TIGIT on ILC2s from HDs and PB−MM pts following 5 days of culture with autologous MM cells. (*n* = 10). (**F**) Representative dot plots and statistical analysis showing Annexin V expression on ILC2s from MM pts (PB and BM). Scatter plots indicate percentage ± SEM of Annexin V+ ILC2s from MM pts (PB and BM); *** *p* < 0.001. (*n* = 20). (**G**) Representative dot plots and relative statistical analysis showing expression of 7−AAD and Annexin V on ILC2s from BM−MM pts following 48 h of culture with primary MM cells in presence of TIGIT−blocking mAb or IgG1 kappa isotype as control. Scatter plots indicate percentage ± SEM of death cells (*n* = 10). *** *p* < 0.001. (**H**) Representative histogram showing expression of DNAM−1 on ILC2s from BM−MM pts following 48 h of culture with autologous MM cells in presence of TIGIT−blocking mAb or IgG1 kappa isotype as control. (**I**) Histograms showing MM cell viability following culture with ILC2s from BM−MM in presence or absence of TIGIT−blocking mAb or IgG1 kappa isotype as control. Scatter plots indicate percentage ± SEM of MM dead cells. ** *p* < 0.01. n.s., not significant; (*n* = 10).

The lowest level of DNAM-1 coupled with no cytotoxic activity raised the question of whether ILC2s from BM might instead express the co-inhibitory counterpart of the DNAM-1 receptor, i.e., TIGIT [21]. TIGIT-expressing ILC2s have been recently described in a model of chronic allergy disease [22], but data related to their expression in cancer have not be reported. As shown in Figure 3A, TIGIT expression was detected in the BM ILC2s from MM pts, whereas it was absent in matched PB ILC2s. TIGIT+ ILC2s were positive for the inhibitory receptor PD-1 and shown to produce the immunosuppressive cytokine IL-10, thus being consistent with an exhausted-like phenotype (Figure 3B). Because their frequency correlated with that of MM cells (Figure 3C), we investigated whether tumor cells might have a role in the induction of this dysfunctional phenotype. Co-culturing ILC2s from HDs with primary MM cells resulted in a gradual reduction in DNAM-1 expression and concomitant TIGIT acquisition (Figure 3D). This acquisition was more pronounced after co-culturing PB ILC2s from MM patients with matched MM cells (Figure 3E), suggesting that they might undergo a certain degree of overstimulation in vivo. In acute myeloid leukemia pts, TIGIT-expressing T cells exhibited a considerable level of apoptotic marker Annexin V [23]. In line with this, TIGIT marks a population of short-lived ILC2s, eliminated by CD155-expressing macrophages during chronic allergic disease [22]. Ex vivo analysis of Annexin V expression revealed that ILC2s from BM but not PB displayed a significant level of this apoptotic marker, which was confined to TIGIT+ ILC2s (Figure 3F). Remarkably, MM cells induced the death of ILC2s via TIGIT, as demonstrated by blocking TIGIT with an antagonistic antibody (Figure 3G). Thus, upon the blockade of TIGIT, ILC2s were less susceptible to apoptosis and showed significant recoveries in DNAM-1 expression (Figure 3H). Accordingly, the cytolytic ability of ILC2 from BM was restored in the presence of TIGIT-blocking antibody, as demonstrated by a decrease in MM cell viability in culture (Figure 3I). Taken together, our data demonstrated that MM cells induce TIGIT expression in ILC2s, shifting their fate toward cell death.

### 3.4. ILC2s Acquire TIGIT and Decrease GZMB Expression Along the Progression of MM

**Figure 4:**
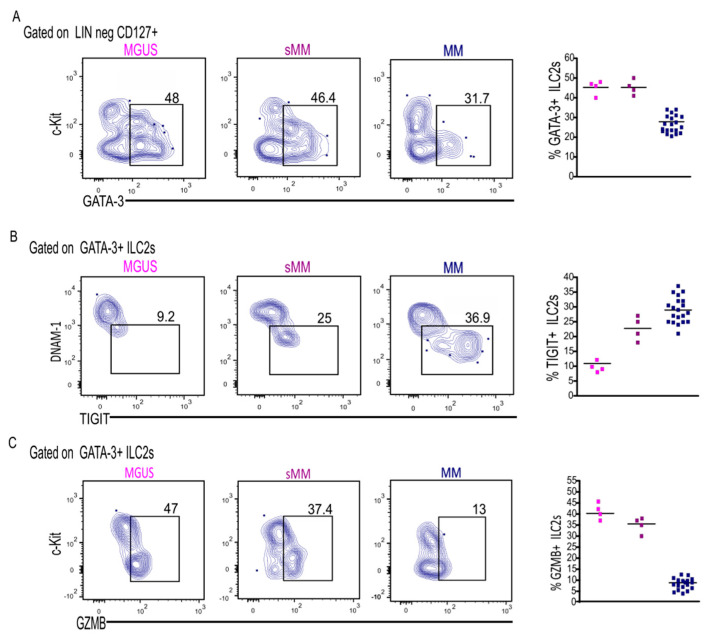
Features of MM-ILC2s along disease progression: (**A**) Representative dot plots showing frequency of GATA−3+ ILC2s in BM of MGUS (*n* = 4), sMM (*n* = 4) and MM pts (*n* = 20). Scatter plot indicates percentage ± SEM of GATA-3+ ILC2s. (**B**) Representative dot plots showing expression of TIGIT on GATA−3+ ILC2s in BM during MM progression. Scatter plot represents percentage ±SEM of TIGIT+ ILC2s. (**C**) Intracellular expression of GZMB on GATA−3+ ILC2s in BM of MGUS (*n* = 4), sMM (*n* = 4) and MM pts (*n* = 20). Scatter plot indicates percentage of GZMB+ ILC2s. Scatter plots: MGUS-pink; sMM-violet; MM-blue.

MM is often preceded by precursor conditions, namely monoclonal gammopathy of undetermined significance (MGUS) and smoldering Multiple Myeloma (sMM) [14]. Since previous studies reported the capability of the immune system to recognize these earliest lesions, we analyzed whether ILC2 alterations, such as their reduced frequency and cytolytic/exhausted profile detected in MM pts, were already present in premalignant lesions. Interestingly, these modifications were already present in MGUS patients, and, specifically, ILC2 frequency was slightly reduced in MGUS and sMM pts, reaching its lowest levels in MM pts (Figure 4A). Similarly, DNAM-1 expression progressively decreased in ILC2s along disease progression (Figure 4B). This decrease was accompanied by the appearance and/or increase of the “overactive” TIGIT+ ILC2s, most likely due to the presence of malignant MM cells (Figure 4B). Of note is that the frequency of GZMB+ ILC2s was significantly higher in MGUS, compared with that in MM, and decreased during sequential stages of disease, supporting the stronger cytotoxic potential of these cells in precursor conditions (Figure 4C). Figure 4 shows the modifications to ILC2 compartments progressively occurring along the disease’s evolution in a representative patient followed-up during progression from MGUS to MM.

## 4. Discussion

ILC2s, similarly to other ILC subsets, are highly plastic. The transition from a steady state, able to maintain tissue homeostasis, to an activated state is influenced by cytokines present in inflamed and cancerous tissues, which allow ILC2s to act either directly or indirectly on tumor immunity [19,24,25]. The heterogeneous expression of c-Kit on ILC2s is a regulator of plasticity in these cells, enabling them to adapt their functions to specific demands of the immune response. ILC2s with high c-Kit expression also express RORγt and secrete IL-17, which are common features of ILC3s and mainly localized in the skin, thymus, blood, tonsil, and intestine [26,27,28]. c-Kit^lo^ ILC2s mainly produce type 2 cytokines and are found in the blood, tonsil and intestine [27,28]. Here, we observed that activating receptors involved in the recognition of tumor cells are also differently expressed in these subsets of ILC2s. While NKG2D and NKp30 were mainly confined to c-Kit^hi^ ILC2s, DNAM-1 was present on all ILC2s, and its level was significantly higher on c-Kit^lo^ ILC2s. By analyzing different isoforms of NKp30, including the immune-stimulatory NKp30a and NKp30b and immuno-regulatory NKp30c isoforms, Salimi et al. showed that ILC2s expressed only the last one, and, accordingly, K562-mediated cross-linking of NKp30 receptor induced increased production of IL-13, which is involved in the activation of immunosuppressive cells [29]. Similarly, an increase in NKG2D in ILC2s has been associated with elevated type 2 cytokines [30]. However, their upregulation occurred when ILC2s were exposed to IL-18, and to a lesser extent to IL-2, but not to IL-33, suggesting an association between NKG2D+ ILC2s and NK/ILC1-promoting conditions [30]. Indeed, an analysis of c-Kit^hi^ ILC2s plasticity revealed an early commitment toward ILC1s and delayed differentiation toward the ILC3 phenotype [20]. In this regard, NKG2D could play a role in the induction of the cytotoxic molecule GZMB in ILC2s, typically expressed by NK cells. However, the contribution of NKG2D appear less relevant compared to that of DNAM-1 [13]. This is in agreement with the observation that GZMB expression is significantly higher in c-Kit^lo^ cells, where NKG2D is largely absent and DNAM-1 shows the highest expression. Compared to HD, in MM patients, the ILC2s of PB expressed a lower level of the DNAM-1 receptor but a considerable level of GZMB, enabling them to kill MM cells (Figure 2B,C,G). In BM, on the other hand, a drastic downregulation of DNAM-1 was associated with the impaired expression of GZMB and concomitant acquisition of the co-inhibitory receptor TIGIT. TIGIT represents a potent inhibitor of myeloma-specific immunity, and these results reveal a novel mechanism of action through which this immune checkpoint inhibitor can favor tumor escape [31,32,33]. Indeed, in our experimental setting, TIGIT blocking restored both the DNAM-1 expression and cytolytic ability of BM-ILC2s against malignant plasma cells. However, this relevant phenomenon should be confirmed in a large cohort of pts as well as in an in vivo experimental model of MM. Concurrently, TIGIT+ ILC2s expressed PD-1, which has been reported to dampen ILC2-dependent antitumor activity [9,10]. In human melanoma, PD-1 blockade leads to the accumulation of ILC2s within the tumor, an event associated with the better survival of patients [9]. Hence, it is conceivable that targeting TIGIT in association with PD-1 blockade could represent a potential synergistic approach to harnessing ILC2 functions for antitumor immunotherapies. In this regard, a reliable protocol capable of expanding human ILC2s with high antitumor potential was recently developed and their infusion significantly delayed tumor growth in vivo [13]. In light of our results, a possible way to optimize this protocol, and thus improve its efficacy, might be monitoring the presence of and eventually exclude dysfunctional ILC2-expressing TIGIT before the adoptive transfer of cytotoxic ILC2s. In conclusion, our current results represent progress in the understanding the cytolytic immune effector cells involved in the anti-MM response. We uncovered a novel mechanism of tumor escape from ILC2-mediated immune surveillance, providing further rationale for the clinical employment of anti-TIGIT mAb in the treatment of these pts, and, potentially, in the earlier stages of MM too, with the aim of slowing down disease progression. This information might also be useful for designing novel and more effective ILC2 cell-based immunotherapeutic strategies for MM disease.

## 5. Conclusions

The ability of ILC2s to acquire unique characteristics or adapt to local environmental cues is an attractive paradigm because it potentially enables the manipulation of them for the purposes of treatment. DNAM-1 triggering and stimulation with specific cytokines, including IL-15 and IL-2, represent key determinants of ILC2-mediated antitumor activity. On the other hand, these stimuli maintain the ability of ILC2 to respond and secrete type 2 cytokines, typically associated with a pro-tumor microenvironment. These findings highlight the dual nature of this innate population, which might have important implications in therapeutic protocols involving these cells. Thus, a deeper understanding of ILC2 biology, particularly the elucidation of their regulome, is important for providing insights into the genomic mechanisms that specify different functions and for guiding the future development of ILC2-based therapeutic methods.

## Data Availability

The data supporting the findings of this study are available from the corresponding author, GF, upon reasonable request.

## Supplementary Materials

The following supporting information can be downloaded at https://www.mdpi.com/article/10.3390/cancers17020263/s1, Figure S1. Gating strategy to identify ILC2s and c-Kit^hi^ ILC2 subset distribution in HD and MM pts. Figure S2. GZMB expression in ILC2 subsets. Table S1. Clinical parameters of MGUS, sMM and MM patients. Table S2. List of antibodies used in the study.
