# Supplementary material for: Multiple Myeloma Cells Shift the Fate of Cytolytic ILC2s Towards TIGIT-Mediated Cell Death
Source: Cancers (Basel). 2025 Jan 15;17(2):263. doi: 10.3390/cancers17020263 (PMC11763689; doi:10.3390/cancers17020263)
Supplement: Supplementary file 1 [file cancers-17-00263-s001.zip › cancers-3371466-supplementary.pdf]

## Supplemental Material

# Multiple Myeloma Cells Shift the Fate of Cytolytic ILC2s Towards TIGIT-Mediated Cell Death

**FFabiana Drommi [1,†], Alessia Calabrò [1,†], Gaetana Pezzino [1], Grazia Vento [2], Josè Freni [3], Gregorio Costa [1,4], Riccardo Cavaliere [1,4], Irene Bonaccorsi [1], Alessandro Allegra [5], Guido Ferlazzo [2,6,*], Claudia De Pasquale [1,‡] and Stefania Campana [1,‡]**

1 Laboratory of Immunology and Biotherapy, Department Human Pathology in Adulthood and Childhood "Gaetano Barresi", University of Messina, 98125 Messina, Italy; drommifabiana@gmail.com (F.D.); calabroalessia@hotmail.it (A.C.); tpezzino@unime.it (G.P.); gregorio.costa@unime.it (G.C.); rcavaliere@unime.it (R.C.); ibonaccorsi@unime.it (I.B.); cdepasquale@unime.it (C.D.P.); scampana@unime.it (S.C.)

2 Department of Experimental Medicine (DIMES), University of Genoa, 16132 Genova, Italy; grazia.vento@edu.unige.it

3 Laboratory of Histology, Department of Biomedical, Dental, Morphological and Functional Imaging Sciences, University of Messina, 98125 Messina, Italy; jofreni@unime.it

4 Clinical Pathology Unit, University Hospital Policlinico "G. Martino", 98125 Messina, Italy

5 Division of Hematology, Department of Human Pathology in Adulthood and Childhood "Gaetano Barresi", University of Messina, 98125 Messina, Italy; alessandro.allegra@unime.it

6 Unit of Experimental Pathology and Immunology, IRCCS Ospedale Policlinico San Martino, 16132 Genova, Italy
*Correspondence: guido.ferlazzo@unige.it

†These authors contributed equally to this work and share first authorship.

‡These authors contributed equally to this work and share last authorship.

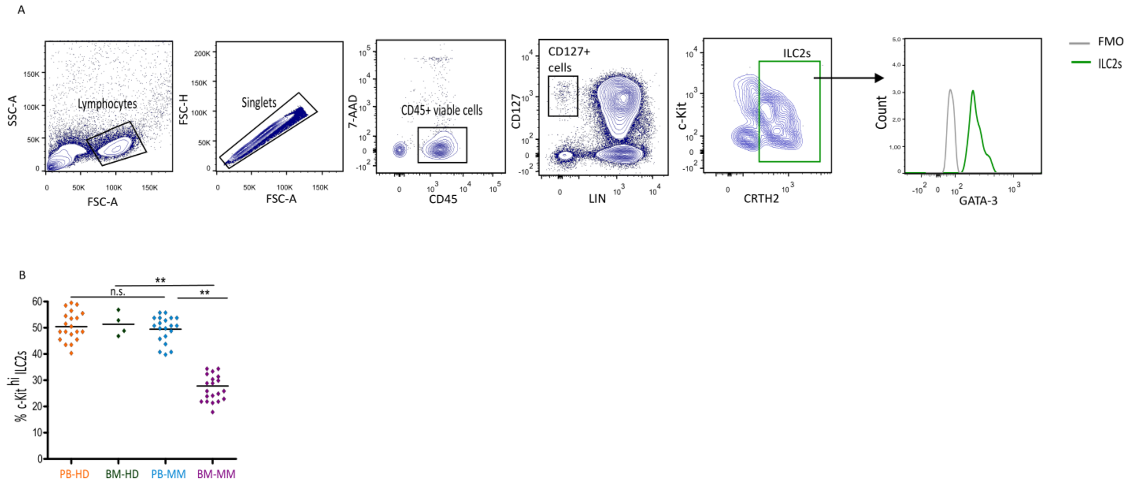


**Figure S1: Gating strategy to identify ILC2s and c-Kit$^{hi}$ ILC2 subset distribution in HD and MM pts.**
(A) Representative gating strategy to identify ILC2s (7-AADneg, single cells, CD45+ LIN neg CD127+ c-Kit $^{hi/lo}$ CRTH2+ GATA-3+) from PB -HD. (B) Statistical analysis shows percentage of c-Kit$^{hi}$ ILC2s from PB (n=20) and BM (n=4) of HD and MM pts (n=20). Scatter plot showing the percentage of c-Kit$^{hi}$ ILC2s from PB and BM of HD and MM pts. n.s. not significant. ** p<0.01.

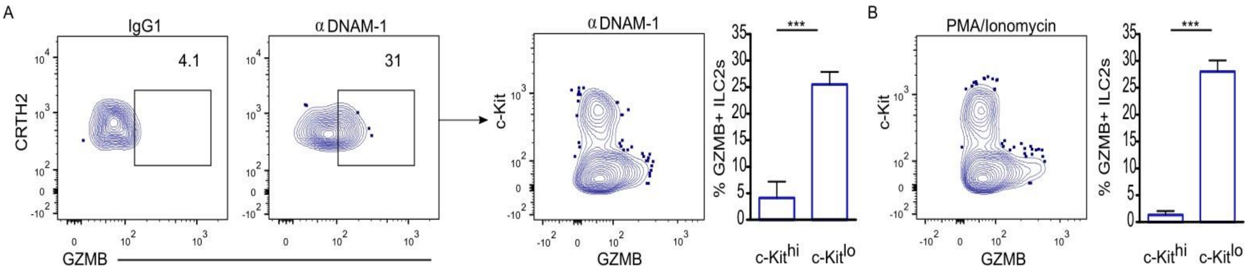


**Figure S2. GZMB expression in ILC2 subsets**

(A) Expression of GZMB assessed on PB-ILC2s from HD following triggering of DNAM-1 or (B) 3h stimulation with PMA/Ionomycin. Bars represent percentage ±SEM of GZMB+ in c-Kit$^{hi}$ and c-Kit$^{lo}$ ILC2s *** $p < 0.001$.

**Table S1. Clinical parameters of MGUS, sMM and MM patients.**

| Patient No. | Age,y /sex | Diseas e | Clinical stage (ISS) | Monoclonal Ig |
|---|---|---|---|---|
| 1 | 60/M | MM | III | IgG κ |
| 2 | 65/M | MM | I | IgG κ |
| 3 | 59/M | MM | III | IgG κ |
| 4 | 60/F | MM | I | IgG κ |
| 5 | 65/F | MM | III | IgA κ |
| 6 | 67/F | MM | III | IgG κ |
| 7 | 62/F | MM | II | IgG κ |
| 8 | 60/M | MM | III | IgG κ |
| 9 | 54/F | MM | I | IgG κ |
| 10 | 65/M | MM | I | IgG κ |
| 11 | 60/F | MM | II | IgG κ |
| 12 | 62/M | MM | II | IgA κ |
| 13 | 58/F | MM | I | IgG λ |
| 14 | 65/M | MM | II | IgG κ |
| 15 | 70/M | MM | I | IgG κ |
| 16 | 62/F | MM | III | IgA κ |
| 17 | 65/F | MM | III | IgG λ |
| 18 | 63/F | MM | II | IgA κ |
| 19 | 64/M | MM | III | IgG λ |
| 20 | 60/F | MM | III | IgG λ |
| 21 | 58/F | sMM | n/a | IgG λ |
| 22 | 54/M | sMM | n/a | IgG κ |
| 23 | 55/M | sMM | n/a | IgG κ |
| 24 | 60/F | sMM | n/a | IgG κ |
| 25 | 52/M | MGUS | n/a | IgG κ |
| 26 | 50/M | MGUS | n/a | IgG λ |
| 27 | 47/F | MGUS | n/a | IgA κ |
| 28 | 62/M | MGUS | n/a | IgG κ |

-F, female; M, male; ISS, International Staging System; sMM, smoldering MM; MGUS, monoclonal gammopathy of undetermined significance; n/a, not applicable.

**Table S2. List of antibodies used in the study**

| mAbs | Clone | Fluorochrome | Distributors |
|---|---|---|---|
| CD3 | UCHT1 | FITC | Beckam Coulter |
| CD45 | J.33 | BV480 | Beckam Coulter |
| CD14 | RMO52 | FITC | Beckam Coulter |
| CD19 | 89B (B4) | FITC | Beckam Coulter |
| CD117 | 104D2D1 | ECD | Beckam Coulter |
| CRTH2 | BM16 | PE | Biolegend |
| CD62L | DREG56 | PeCy5.5 | Beckam Coulter |
| NKp80 | REA845 | FITC | Miltenyi Biotec |
| CD127 | R34.34 | PeCy 7 | Beckam Coulter |
| NKp30 | Z25 | PE | Beckam Coulter |
| DNAM-1 | 11A8 | PE | Biolegend |
| NKG2D | ON72 | APC | Beckam Coulter |
| Granzyme B | GB11 | FITC | BD Biosciences |
| CD161 | 191B8 | PE | Beckam Coulter |
| 7-AAD | | BB700 | Beckman Coulter |
| CD25 | B1.49.9 | APC-Alexa Fluor 700 | Beckman Coulter |
| KLRG1 | 2F1 | Brillant Violet 785 | Biolegend |
| IL-10 | JES5-16E3 | PE | Biolegend |
| IL-13 | JES10-5A2 | PE | Biolegend |
| PD-1 | PD1.3 | PeCy7 | Beckman Coulter |
| CD138 | B-A38 | PeCy5.5 | Beckman Coulter |
| CD38 | LS198-4-3 | BV421 | Beckman Coulter |
| GATA-3 | 16E10A23 | APC | Biolegend |
| PVR (CD112) | L95 | | |
| Nectin-2 (CD155) | L14 | | n.a. |
| IgG1 isotype | n.a. | | Invitrogen |
| IgM isotype | n.a. | | Invitrogen |
| TIGIT | 741182 | BUV395 | BD Biosciences |
| DNAM-1 Ab | F5 and F22 | | |
| TIGIT Ab | | | MedChemExpress |
| IgG1 kappa isotype | | | MedChemExpress |

n.a. = not available.
